# 

**Published:** 1867-11

**Authors:** 


### BIBLIOGRAPHICAL NOTICES.

---

*The Tree of Life; or Human Degeneracy; its Nature and Remedy.* By Isaac Jennings, M. D. Publishers, Miller, Wood & Co., New York. A small treatise on the treatment of human life on Orthopathic principles, including directions for the treatment of human physical life. Part 1st. is devoted to Man's Spiritual Degeneracy; Part 2nd. to Man's Physical Degeneracy, its nature and remedy. We take the following from page 220:

"There are what may aptly be called critical periods in slender human life, from infancy to advanced age, growing out of new developements or essential changes called for at different stages of life's progress. Teething forms one of these changes." "After a few months, many children have trouble with their teeth, and the reason of it is obvious. Teeth cost something." "There must be a special outlay of vital force to bring them forward, and there is not enough in store in the depository pertaining to the group of organs concerned in teeth making for the purpose without curtailing appropriations to other organs; and these parts are thereby left so deficient in sustaining energy that they falter in their action; and this faltering is manifested by various phenomena called symptoms." "Patient and careful nursing is all that is called for on the part of the mother." "Keep the babe well covered with flannel, let it have the breast at suitable intervals, and as you value the future welfare of the child, eschew "Winslow's soothing syrup," and all such baby quieters. As the gum swells over a protruding tooth, it may be rubbed gently, occasionally, with any smooth hard substance, and as the tooth nears the surface, the gum over it may be divided."

*A Dictionary of Medical Terminology, Dental Surgery, and the Collateral Sciences.*—By Chapin A. Harris M.D., D.D.S. Third edition, carefully Revised and Enlarged, by Ferdinand J. S. Gorgas, M.D.,D.D.S.—Philadelphia, Lindsay and Blakiston, 1867.

Dr. Harris' Dictionary has been so long before the profession, and has been received with such universal favour that it is idle to say anything about its merits. It is not, however, irrelevant to inquire how the new edition differs from the old.

It may surprise some to see the announcement on the title page "Revised and *Enlarged*" and then to ascertain that the new edition contains only 743 pages while the old had 800. Yet in spite of the arithmetical anomoly, the title page tells the literal truth. It has now been twelve years since the second edition was issued from the press. In that time great changes have taken place. The rapid advance of all the sciences which have furnished terms to this book has necessarily rendered obsolete many of the words then in common use. These have been dropped, and room thus made for addition of others which the same advance has rendered absolutely necessary to express the received opinions of the day. A large amount of space has been gained by omiting many articles which had been copied from the author's "Principles and Practice of Dental Surgery," and substituting for them simple references to that volume. No one who pretends to possess a Dental library is without both these books, so that nothing is lost by this excision. By thus economizing space the Editor has been enabled to diminish the bulk by nearly sixty pages while he has enlarged its contents by two or three thousand words.

Dr. Gorgas has performed his task diligently, and conscientiously. In the comparison which we have been enabled to make of the two editions, we have not detected the omission of a single word which we deemed it important to retain. Nor does he appear to have committed the opposite fault, so tempting to an editor, of crowding in a number of terms of no practical value whatever, and only serving, like the countermarches of a skilful strategist, to make an imposing display and conceal the real poverty of his resources. On the contrary, the new words, as far as our observation goes, are legitimate terms, regularly if not frequently used. He has followed the progress of modern science and given us the last results of its terminology, so far as they have any bearing upon Dental Surgery. Of course, the minute technicalities of the allied sciences are not given; otherwise three such volumes as this would prove insufficient.

The typographical execution of the book is generally good. We have however, noticed a few errors both in typography and quantity, the latter probably due to the printer. P.

---

*The Medical Gazette.*—This new candidate for professional favour is published by A. Simpson and Co., No., 60 Duane Street, New York. We have received the first number, dated September 28th. It is strictly speaking, a Medical newspaper, giving all items of intelligence likely to prove interesting to physicians, whether engaged or not in active practice. Besides these, it also contains letters of distinguished teachers of medicine, abstracts of proceedings of Societies, and Hospital reports.

We are very much pleased with this specimen number. The matter is interesting and valuable, and the mechanical getting up of the paper is unexceptionable. It is published at the very moderate sum of two dollars a year which ought to bring it within the means of the poorest.

---

